# Melatonin, an endogenous hormone, modulates Th17 cells via the reactive-oxygen species/TXNIP/HIF-1α axis to alleviate autoimmune uveitis

**DOI:** 10.1186/s12974-022-02477-z

**Published:** 2022-05-27

**Authors:** Jun Huang, Zhuang Li, Yunwei Hu, Zuoyi Li, Yanyan Xie, Haixiang Huang, Qian Chen, Guanyu Chen, Wenjie Zhu, Yuxi Chen, Wenru Su, Xiaoqing Chen, Dan Liang

**Affiliations:** State Key Laboratory of Ophthalmology, Zhongshan Ophthalmic Center, Guangdong Provincial Key Laboratoryof Ophthalmologyand VisualScience, Sun Yat-Sen University, Guangzhou, 510060 China

**Keywords:** Experimental autoimmune uveitis, Melatonin, Th17/Treg, Reactive-oxygen species, TXNIP, HIF-1α

## Abstract

**Background:**

Melatonin, an indoleamine produced by the pineal gland, plays a pivotal role in maintaining circadian rhythm homeostasis. Recently, the strong antioxidant and anti-inflammatory properties of melatonin have attracted attention of researchers. We evaluated the therapeutic efficacy of melatonin in experimental autoimmune uveitis (EAU), which is a representative animal model of human autoimmune uveitis.

**Methods:**

EAU was induced in mice via immunization with the peptide interphotoreceptor retinoid binding protein 1–20 (IRBP_1–20_). Melatonin was then administered via intraperitoneal injection to induce protection against EAU. With EAU induction for 14 days, clinical and histopathological scores were graded to evaluate the disease progression. T lymphocytes accumulation and the expression of inflammatory cytokines in the retinas were assessed via flow cytometry and RT-PCR, respectively. T helper 1 (Th1), T helper 17 (Th17), and regulatory T (Treg) cells were detected via flow cytometry for both in vivo and in vitro experiments. Reactive-oxygen species (ROS) from CD4 + T cells was tested via flow cytometry. The expression of thioredoxin-interacting protein (TXNIP) and hypoxia-inducible factor 1 alpha (HIF-1α) proteins were quantified via western blot.

**Results:**

Melatonin treatment resulted in notable attenuation of ocular inflammation in EAU mice, evidenced by decreasing optic disc edema, few signs of retinal vasculitis, and minimal retinal and choroidal infiltrates. Mechanistic studies revealed that melatonin restricted the proliferation of peripheral Th1 and Th17 cells by suppressing their transcription factors and potentiated Treg cells. In vitro studies corroborated that melatonin restrained the polarization of retina-specific T cells towards Th17 and Th1 cells in addition to enhancing the proportion of Treg cells. Pretreatment of retina-specific T cells with melatonin failed to induce EAU in naïve recipients. Furthermore, the ROS/ TXNIP/ HIF-1α pathway was shown to mediate the therapeutic effect of melatonin in EAU.

**Conclusions:**

Melatonin regulates autoimmune T cells by restraining effector T cells and facilitating Treg generation, indicating that melatonin could be a hopeful treatment alternative for autoimmune uveitis.

**Supplementary Information:**

The online version contains supplementary material available at 10.1186/s12974-022-02477-z.

## Background

Melatonin is an endogenous hormone synthesized mainly by the pineal gland [1] and the retina [2], and has antioxidant and anti-inflammatory effects [3, 4]. Previous studies have demonstrated that melatonin acts as an anti-inflammatory through a variety of mechanisms, such as, suppressing the progression of diabetic retinopathy via inhibition of p38/TXNIP/NF‐κB pathway [5]; Preventing Th17/Treg imbalance through activation of the AMPK/SIRT1 pathway to ameliorate necrotizing enterocolitis [6]. In experimental autoimmune encephalomyelitis (EAE) model, melatonin can increase IL-10 expression and suppress chemotaxis to inhibit inflammation and reduce the severity of EAE [7]. Farez et al. reported that melatonin blocked the differentiation of pathogenic Th17 cells as well as induced the generation of Tr1 cells via Erk1/2 and activated the IL-10 promoter by ROR-α, in addition, melatonin modulated the differentiation of human multiple sclerosis (MS)Th17 and Tr1 cells in vitro [8]. Previous study has shown that Melatonin can reduce peripheral and central Teff responses, as well as enhance Treg frequency and IL-10 synthesis in the central nervous system, contributing to EAE [9]. However, the therapeutic effects of melatonin in EAU have not yet been elucidated.

Autoimmune uveitis (AU) is a potentially sight threatening intraocular inflammatory process accounting for nearly 10% of cases of severe visual impairment worldwide, and 25% of patients of legal blindness in the developing world [10–12]. There are many pathological changes in patients with AU, characterized by retinal vasculitis, optic nerve damage, and photoreceptor damage. The current treatment modalities, including biologics and immunosuppressants, are not specific and do not cure the disease. Furthermore, they are often associated with severe adverse effects. Hence, the safe and effective alternatives are needed urgently for AU.

EAU is an established animal model of human uveitis caused by interphotoreceptor retinoid binding protein (IRBP) specific effector T cells (Teff) [13]. Pathogenic mechanism of AU was undetermined. Studies revealed that increased Teff cells and/or decreased Treg cells mediated initiation and progression of AU [14–16]. Thus, suppressing the polarization of Th1 and Th17 cells, and/or potentiating Treg cells are supposed to attenuate AU.

In the current study, we investigated the therapeutic efficacy of melatonin in AU and explored its potential mechanism of action. We demonstrated that systemic administration of melatonin alleviated EAU by decreasing the ratios of Th1 and Th17 effector cells and increasing the ratio of Treg cells, both locally and peripherally. Mechanistic investigations revealed that melatonin restricted the polarization of retina-specific T cells by downregulating RORγt via the ROS/ TXNIP/HIF-1α pathway. Our data indicated that melatonin directly regulated autoimmune responses, and supported potential as an alternative therapy option for human AU.

## Materials and methods

### Mice

C57BL/6J mice (female, 6–8 weeks old; 20 ± 1.5 g) from the Guangzhou Animal Testing Center (Guangzhou, China) were kept in specific pathogen-free conditions. The Institutional Animal Care and Use Committee of Zhongshan Ophthalmic Center Sun Yat-sen University approved this study, which was performed in compliance with the ARVO Statement for the Use of Animals in Ophthalmic and Vision Research.

### EAU induction and treatment with melatonin

Female mice were subcutaneously immunized with IRBP_1–20_ (amino acid sequence: GPTHLFQPSLVLDMAKVLLD; GL Biochem, Shanghai, China) emulsified in Freund’s Complete Adjuvant (Difco, Detroit, MI, USA) containing *Mycobacterium tuberculosis* strain H37Ra (Difco; 1:1 v/v). On day 0 and day 2, pertussis toxin (Sigma-Aldrich, St. Louis, MO, USA) was administered intraperitoneally [17, 18]. The above methods have also been introduced in detail in our previous study [19]. From the third day of immunization, melatonin (Sigma-Aldrich) was administered intraperitoneally (10 mg/kg or 80 mg/kg in solution that contains 4% DMSO and 96% PBS). Control group mice were treated with the same volume of vehicle instead of melatonin.

### Fundoscopy and histology study in EAU

On day 14 after immunization, the fundus of the mice was examined by Micron IV Retinal Imaging Microscope (PHOENIX, USA) and scored clinically. Clinical scoring was based on the previous study, graded 0 ~ 4, evaluated by retinal vasculitis, choroidal and retinal infiltration/lesions, papilledema, retinal hemorrhage, retinal detachment, retinal atrophy etc. [18]. On day 14, the mice were euthanized, and eyes were enucleated and stored in 4% neutral buffered formalin solution for 24 h at room temperature. Then, samples were dehydrated, embedded and hematoxylin and eosin (H&E) stained. Histopathological changes were evaluated and graded in accordance with previously described criteria, included inflammatory cell infiltration, retinal folds and detachments, granulomas in choroid and retina, diffuse retinal detachment with serous exudate, extensive photoreceptor cell damage, subretinal neovascularization, etc. [18].

### Retina-infiltrating cells isolation

This method was consistent with our previous published paper [19]. Briefly, Retinas from eyes were dissected and then incubated with collagenase D (1 mg/mL, Roche, Switzerland) and DNase I (100 µg/mL, Sigma-Aldrich) in10% Fetal Bovine Serum (FBS) supplemented RPMI-1640 culture medium (Gibco, USA) at 37 °C for 30 min. Cells were filtered and resuspended. Finally, the infiltrated retinal cells were obtained for flow cytometry analysis.

### Flow cytometry

Cells were stained with surface markers including antimouse CD4 Percp-Cy5.5 (clone GK 1.5), antimouse CD45 Brilliant Violet 510 (clone 30-F11), antimouse CD8α PE (clone 53-6.7), antimouse CD19 Brilliant Violet 650 (clone 6D5), antimouse CD44 APC (clone IM7), antimouse CD25 PE/Cyanine7 (clone 3C7), antimouse CD62L FITC (clone MEL-14), anti-mouse CD279 (PD-1) APC/Cyanine7 (clone 29F.1A12), antimouse CD11c PE (clone N418). Above antibodies are from eBioscience (Carlsbad, CA, USA) or BioLegend (San Diego, CA, USA). For intracellular cytokine IFN-γ and IL-17A, the cells were incubated with ionomycin (500 ng/mL), PMA (50 ng/mL), and BFA (1 µg/mL) (Sigma-Aldrich) for 5 h, and after fixation and permeabilization, the cells were stained with antimouse IL-17A BV650 (clone TC11-18H10.1) and antimouse IFN-γ BV786 (clone XMG1.2). Transcription factor staining kit was used according to the manufacturer's protocol. For the total cellular ROS staining, the cells were incubated with 10 μM 2′,7′-dichlorodihydrofluorescein diacetate (CAS No. 4091-99-0) for 20 min at 37 °C in a cell culture incubator, which were analyzed using a BD LSRFortessa instrument (BD Biosciences, Franklin Lakes, NJ, USA), and the acquired data were processed using FlowJo 10.0 (FlowJo Co., Ashland, OR, USA).

### T cell polarization

Naïve CD4 + T cells (CD4 + CD62L + CD25-CD44-) were isolated from the lymph nodes and spleen of wild type mice using a commercial kit (Miltenyi Biotec, Gladbach, Germany). The naïve CD4 + T cells (purity: 95%; 2 × 10^5^/well) were incubated with anti-CD3/CD28 beads (one bead to five cells) for three days in a 96-well plate. For Treg cell differentiation, the cultures were supplemented with Treg cell differentiation condition [TGF-β1 (10 ng/mL; PeproTech, Rocky Hill, NJ, USA) and recombinant human IL-2 (50 U/mL; PeproTech)].

### Enzyme-linked immunosorbent assay (ELISA)

T cells which were isolated from DLNs of EAU mice were preincubated with melatonin (200 ng/mL) for 4 h, and stimulated with 10 μg/mL IRBP_1-20_ for 3 days. The cell culture supernatants were collected, and the concentrations of IFN-γ and IL-17A were assayed using ELISA kits (Invitrogen, Carlsbad, CA, USA).

### Real-time quantitative polymerase chain reaction (PCR)

Retinas were isolated from the different treatment groups. The total RNA was extracted using TRIzol reagent (Invitrogen) and quantified using a NanoDrop spectrophotometer (ND-1000; NanoDrop Technologies, Wilmington, DE, USA). The total RNA was used to synthesize cDNA using PrimeScript RT Master Mix (Perfect Real Time, TaKaRa Bio Inc., Kusatsu, Japan). Real-time quantitative PCR was performed using SYBR Premix Ex Taq II (TaKaRa Bio Inc.). Glyceraldehyde-3-phosphate dehydrogenase (GAPDH) mRNA was used as an internal control. The relative mRNA expression of IFN-γ, IL-17A, and Foxp3 (Sangon Biotech, Shanghai, China) was analyzed using the 2^–ΔΔCt^ method.

### Western blot assays

Proteins from cultured cells and DLNs were extracted using whole cell lysis buffer (KeyGen Biotech, Jiangsu, China) and measured the concentration of protein according to the protein assay kit’s instructions, which were separated on polyacrylamide-sodium dodecyl sulfate gels, and electro-transferred onto PVDF membrane. Then, blocking with 5% non-fat dry milk, the PVDF membranes were incubated with TXNIP (rabbit; clone D5F3E; Cell Signaling Technology, Danvers, MA, USA), HIF-1α (rabbit; clone D1S7W; Cell Signaling Technology) and GAPDH (rabbit; clone D16H11; Cell Signaling Technology) overnight at 4 °C, followed by incubation with the secondary antibody for 2 h. The western blot signals were imaged using enhanced chemiluminescence (Pierce, Rockford, IL, USA). ImageJ software (NIH, Bethesda, MD, USA) was used to measure the gray scale.

### Adoptive transfer assay to induce EAU

T cells isolated from the DLNs of EAU mice (day 14 after immunization) were stimulated using IRBP_1-20_ (10 μg/mL) under Th17-polarizing conditions, with or without melatonin (200 ng/mL) for 72 h, and were washed three times before administration to C57BL/6J mice (2 × 10^7^ living cells/mouse) via intraperitoneal injection. The extent of retinal inflammation by fundus and HE was evaluated on day 14.

### IRBP-specific responses

In vitro study, the cells (4 × 10^5^) from the DLNs of EAU mice were cultured in 96-well plates with IRBP_1-20_ under Th1-polarizing conditions (ImmunoCult 10953; STEMCELL Technologies, Vancouver, Canada) or Th17-polarizing conditions (anti-IFN-γ [10 μg/mL; R4-6A2], anti-IL-4 [10 μg/mL; L11B11], TGF-β [2.5 ng/mL], IL-6 [25 ng/mL], and IL-23 [10 ng/mL]) in the presence or absence of melatonin (0, 2, 20, or 200 ng/mL). After 3 days, the cells were harvested, and flow cytometry was used for the intracellular inflammatory cytokine.

### Statistical analysis

The data were presented as means ± standard deviation (SD). The Student’s *t* test, Mann–Whitney test, or one-way analysis of variance was selected according to the data sets normality. All data were analyzed by GraphPad Prism 8.2 (GraphPad Software, Inc., La Jolla, CA, USA). *P* < 0.05 was considered to be statistically significant.

## Results

### Melatonin treatment protected mice from retinal injury after EAU induction

**Fig. 1:**
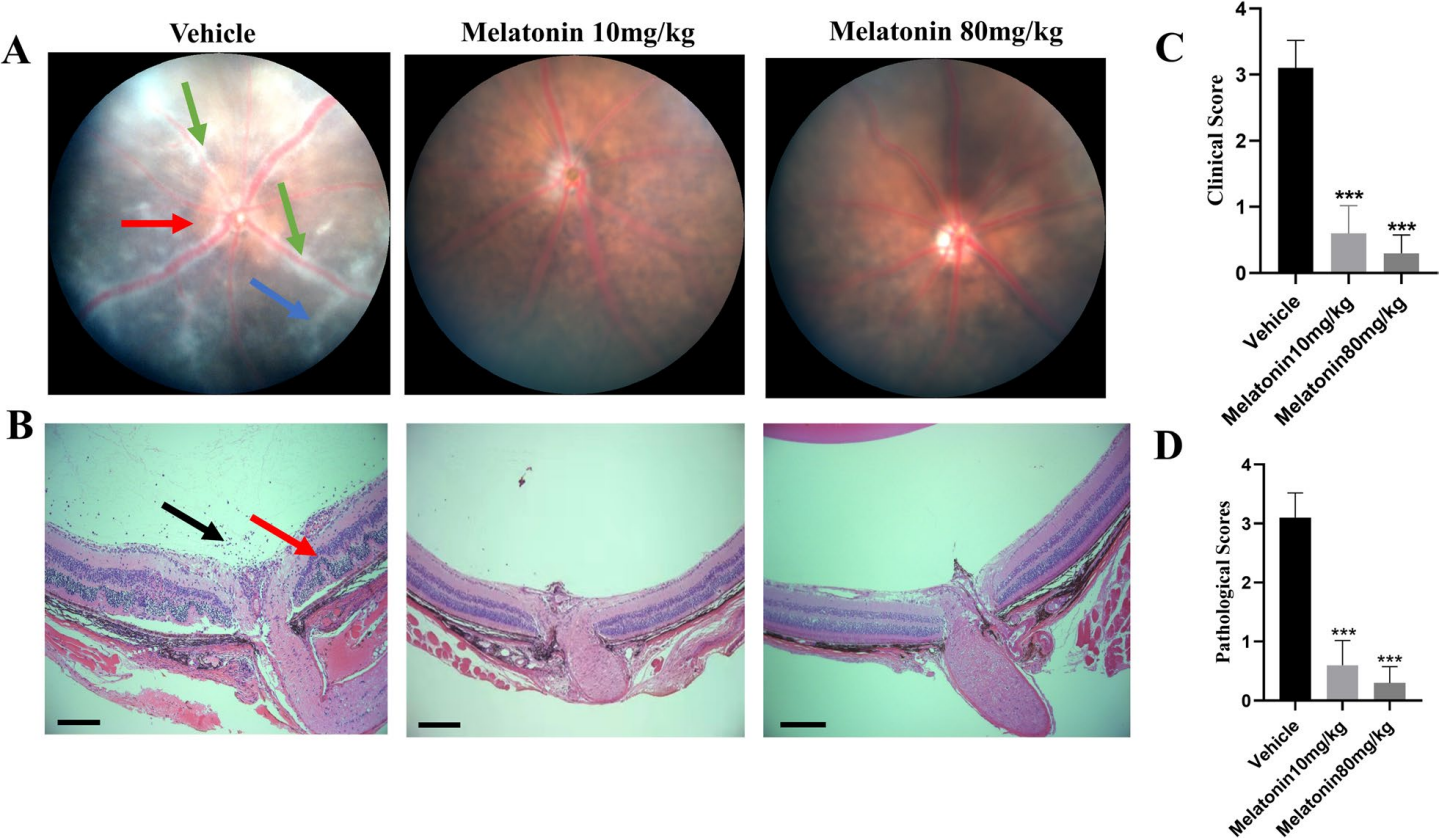
Melatonin treatment protected mice from retinal injury after EAU induction. C57BL/6J mice immunized with IRBP_1-20_ were treated with melatonin at different dosages (0, 10, or 80 mg/kg/day) daily from day three after immunization. **A** Representative pictures of fundoscopic examination in the vehicle group and melatonin (10, 80 mg/kg/day) group at fourteen days after immunization (green arrow: vasculitis, red arrow: papilledema, blue arrow: linear lesions). **B** Representative pictures (H&E) of histopathologic examination in the vehicle group and melatonin (10 or 80 mg/kg/day) group (black arrow: inflammatory cells, red arrow: retinal folding with detachments, scale bars represent 200 μm). **C** The clinical scores of EAU were graded 14 days after immunization (*n* = 5). **D** The pathological scores of EAU via H&E were graded 14 days after immunization (*n* = 5). Representative data from three independent experiments. Significance was determined by one-way ANOVA. ***P < 0.001 vs. vehicle group

Melatonin was administered daily from the third day post-immunization to determine the therapeutic effects on EAU. An intraperitoneal dose of 10 or 80 mg/kg was chosen based on a preliminary study [9]. Systemic toxicity (evaluated by mortality rate, behavior, alterations in weight, liver, kidney, intestinal) was not observed with the chosen dose of melatonin (Additional file 1: Fig. S1). Fundus of EAU mice treated with vehicle revealed severe chorioretinal lesions, infiltrations, vasculitis, and prominent retinal folds. Mice treated with melatonin (10 and 80 mg/kg/day) exhibited less vasculitis, and fewer infiltrated inflammatory lesions (Fig. 1A). Histopathological analyses revealed scattered inflammatory cell infiltrations and extensive retinal folding with detachment in the vehicle group. Intraperitoneal administration of melatonin (10 and 80 mg/kg) considerably decreased retinal inflammatory cells infiltration and folds (Fig. 1B). Melatonin treatment significantly decreased the clinical scores and histopathological scores of mice with EAU compared with the vehicle group (Fig. 1C, D). Further, 80 mg/kg melatonin treatment showed better therapeutic effect and was chosen for subsequent in vivo studies. This observation indicates that melatonin significantly reduced the severity of EAU.

### Melatonin treatment altered intraocular Teff/Treg immune balance

**Fig. 2:**
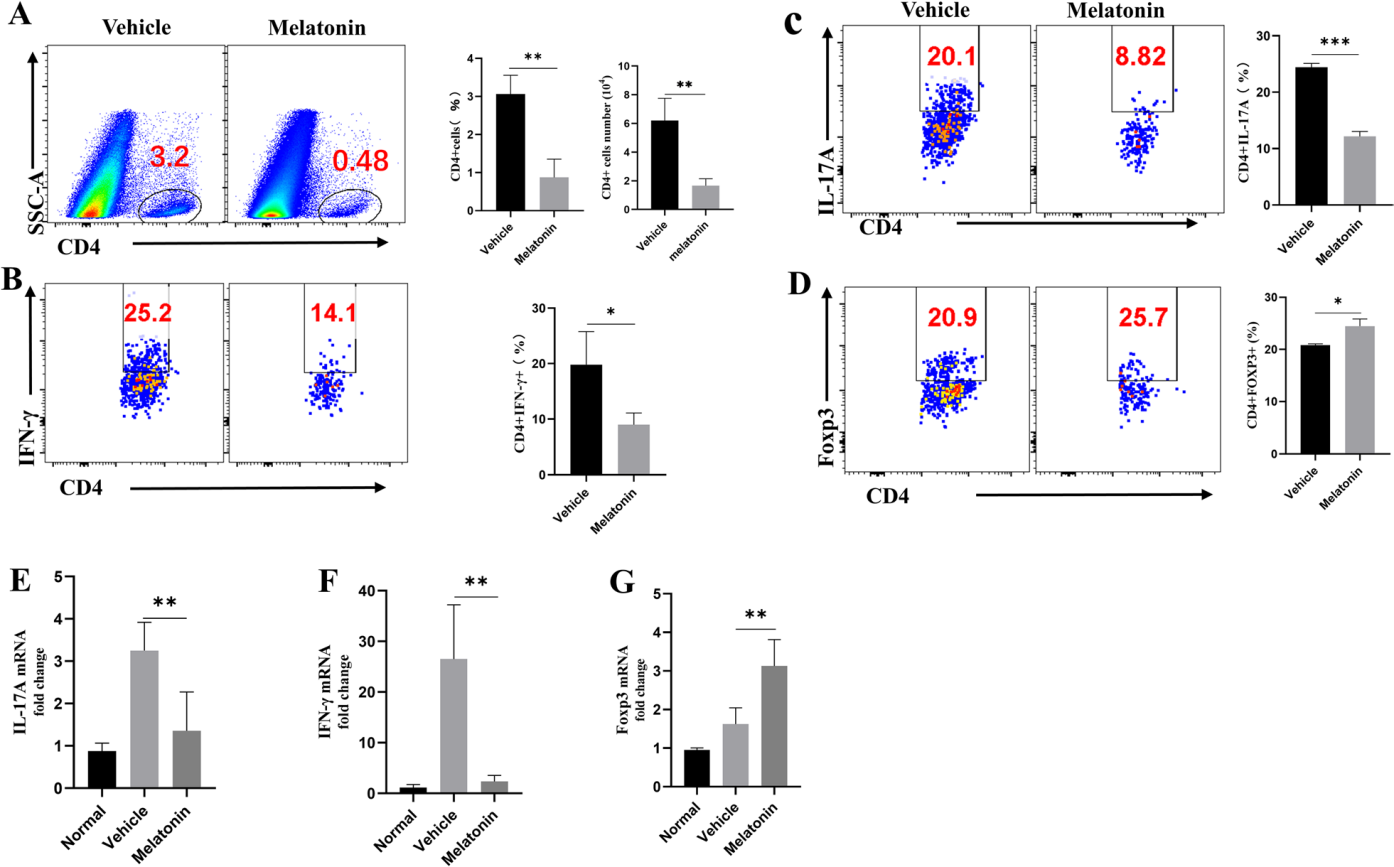
Melatonin treatment altered intraocular Teff/Treg immune balance. **A** The frequency and number of eye-infiltrating CD4 + T cells were revealed by Flow cytometric analysis after melatonin treatment. **B**–**D** Flow cytometric analysis of intracellular expression of interferon gamma (IFN-γ) and interleukin (IL)-17 on CD4 + T cells harvested from the eyes of experimental autoimmune uveitis mice treated with vehicle (dimethyl sulfoxide) or melatonin on day 14 (*n* = 3). **E**–**G** The expression of IL-17A, IFN-γ, and Foxp3 mRNA in the retinas were measured using real-time polymerase chain reaction 14 days after immunization (*n* = 4). The representative values from three independent experiments. Significance was determined by unpaired *t* test (**A**–**D**), or one-way ANOVA (**E**–**G**). *P < 0.05, **P < 0.01, ***P < 0.001

Considering that the active lymphocytes infiltration into the eye is a critical step in retinal inflammation and pathological injury in AU, assays were performed to determine whether melatonin could reduce T lymphocytes aggregation in the eye. Intraocular cells were isolated, stained, and analyzed by flow cytometry 14 days after immunization. Melatonin treatment greatly lessened intraocular CD4 + T cells accumulation compared with treatment with vehicle only (Fig. 2A).

Dysregulated Teff cells and/or Treg cells contribute to the initiation and progression of autoimmune diseases. Therefore, we analyzed the intraocular production of IFN-γ and IL-17A by Th1 and Th17 cells, respectively, to evaluate the effect of melatonin treatment on the CD4 + T cell immune responses. Melatonin treatment significantly decreased the frequency of Th1 and Th17 cells (Fig. 2B, C). Interestingly, melatonin treatment significantly increased the proportion of CD4 + Foxp3 + Treg cells (Fig. 2D). In Additional file 2: Fig. S2, Gating strategy for flow cytometry and total numbers of infiltrating CD45+ cells in the eye were displayed. Subsequently, we investigated the gene expression of IL-17A, IFN-γ, and Foxp3 in the retina using real-time PCR. The results revealed that melatonin markedly suppressed the gene expression of IL-17A and IFN-γ, while increasing the gene expression of Foxp3 (Fig. 2E–G). Overall, melatonin ameliorated retinal inflammation by downregulating intraocular Th17/Th1 cells and upregulating Treg cells.

### Melatonin regulated Th1/Th17 and Treg cell balance in peripheral lymphoid organs of EAU mice

**Fig. 3:**
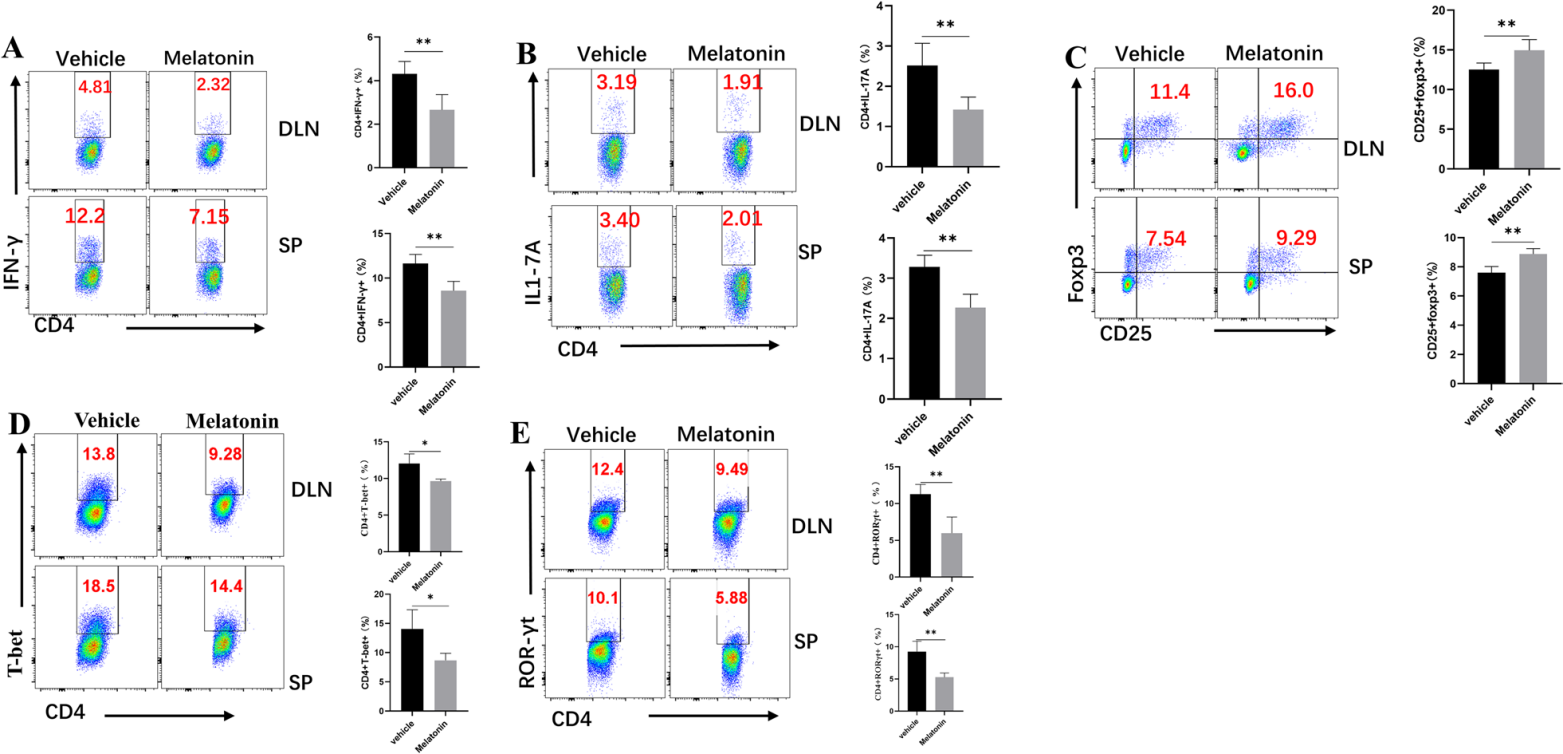
Melatonin regulated Th1/Th17 and Treg cell balance in peripheral lymphoid organs of EAU mice. **A**–**C** The of CD4 + IFN-γ + , CD4 + IL-17A + , and CD25 + Foxp3 + T cells in the DLNs and spleens of EAU mice were evaluated by flow cytometry fourteen days after immunization (*n* = 5). **D**, **E** The lineage transcription factors of Th1 and Th17 in the DLNs and spleens of EAU mice were also analyzed by flow cytometry 14 days after immunization (*n* = 4). The representative data from three independent experiments. Significance was determined by unpaired *t* test (**A**–**E**). **P < 0.01, ***P < 0.001

It is supposed that IRBP-specific T cells are constituted in the DLNs and spleen and migrate through the impaired blood retinal barrier to the retina tissues, resulting in intraocular inflammation and retinal structural destruction. Hence, we evaluated the effects of melatonin on Teff and Treg cells in DLNs and spleen. Single cells were prepared from DLNs and spleen and analyzed by flow cytometry. Melatonin decreased the frequency of Th1 and Th17 cells compared with the vehicle group (Fig. 3A, B). Regulatory T cells were investigated because they are known to suppress inflammation by modulating Teff cells [20]. Melatonin markedly increased the percentage of CD25 + FoxP3 + cells in the DLNs and spleens of EAU mice (Fig. 3C). However, melatonin performed no significant effect on the proportions of Treg proliferation (Additional file 3: Fig. S3). The effects of melatonin on Th1 and Th17 cells were further explored by analyzing their respective transcription factors, T-bet and RORγt. When compared with the vehicle group, melatonin treatment dramatically decreased the levels of T-bet and RORγt (Fig. 3D, E). These data suggested that melatonin treatment could alter the balance of effector T cells and regulatory T cells in periphery, contributing to the inhibition of retinal inflammatory injury.

### Melatonin inhibited IRBP_1-20_-specific Th cell responses and promoted Treg cell differentiation in vitro

**Fig. 4:**
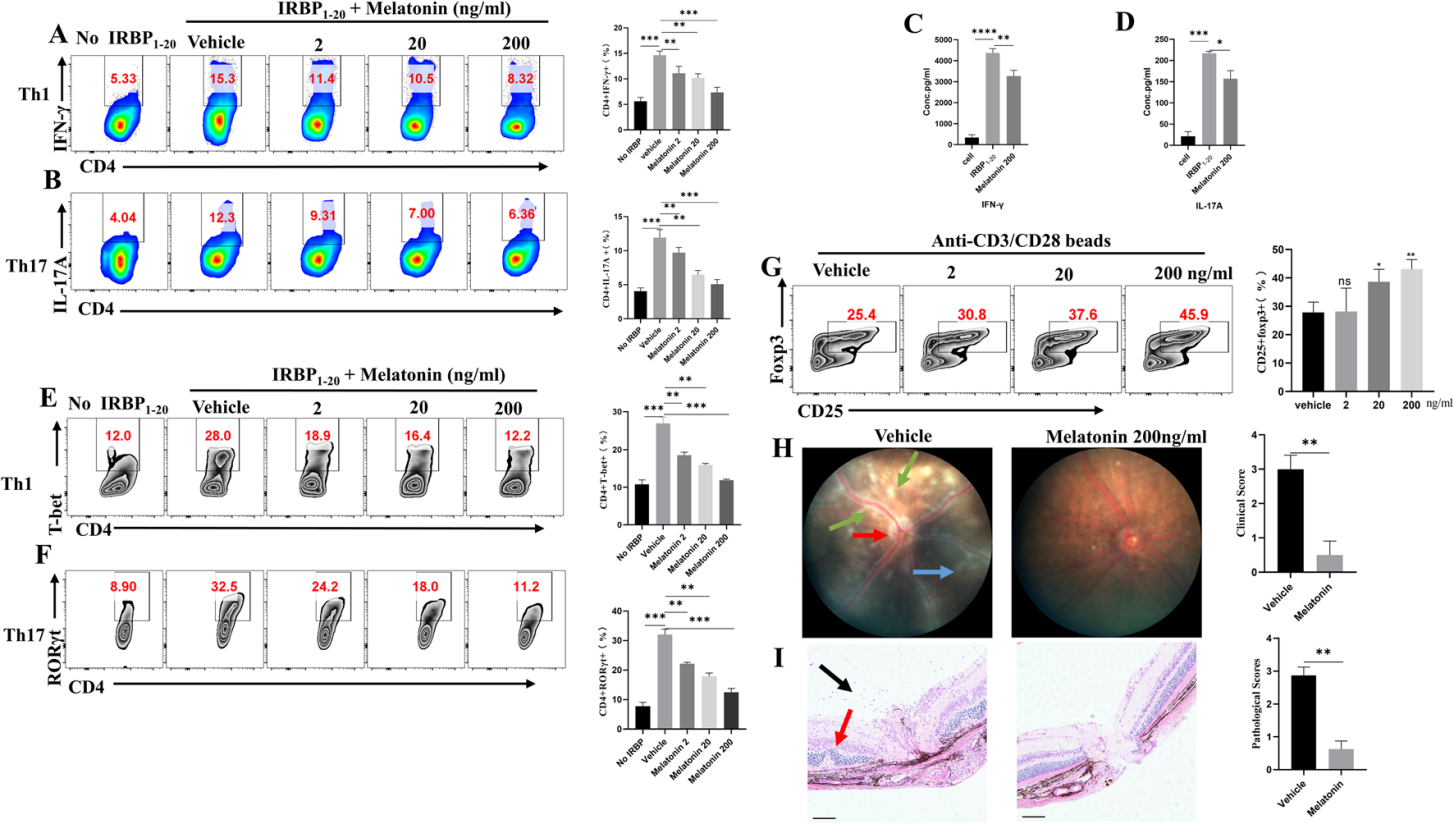
Melatonin inhibited IRBP_1-20_-specific Th cell responses and promoted Treg cell differentiation in vitro. **A**, **B** Cells from DLNs of EAU mice were stimulated with 10 μg/mL IRBP_1-20_ under T helper 1 (Th1) or T helper 17 (Th17)-polarizing conditions with different concentrations of melatonin (0, 2, 20, or 200 ng/mL) for 72 h, the CD4 + cell population was assessed for interferon (IFN)-γ or interleukin (IL)-17A expression by flow cytometry (*n* = 4). **C**, **D** IFN-γ and IL-17A in supernatants were measured via ELISA. **E**, **F** T-bet and RORγt were assayed by flow cytometry (*n* = 4). **G** Naïve CD4 + T cells were polarized to Treg cells with or without melatonin for three days. CD25 + Foxp3 + cells expression was analyzed by flow cytometry (*n* = 4). **H**, **I** Cells were isolated from DLNs of EAU mice and stimulated with IRBP_1-20_ peptide under Th17-polarizing conditions in the absence or presence of melatonin (200 ng/mL). After three days, viable cells were adoptively transferred to C57BL/6J wild-type mice (20 million cells/mouse; *n* = 4). **H** Clinical scores and representative pictures on day 14 (green arrow: vasculitis, red arrow: papilledema, blue arrow: linear lesions). **I** Histology scores and representative pictures of H&E (black arrow: inflammatory cells, red arrow: retinal folding with detachments, scale bars represent 100 μm). The representative data from three independent experiments. Significance was determined by one-way ANOVA (**A**–**G**), or Mann–Whitney test (**H**, **I**). *P < 0.05, **P < 0.01, ***P < 0.001, ****P < 0.0001

To clarify the mechanism(s) by which melatonin regulates Th1 and Th17 cells, we investigated the effects of melatonin on Th1 and Th17 cell differentiation in vitro*.* Cells were obtained from the DLNs of EAU mice and stimulated with IRBP_1-20_ under Th1- or Th17-polarizing conditions, with or without melatonin. The presence of melatonin in the culture system (0, 2, 20, and 200 ng/mL) inhibited the generation of IFN-γ- or IL-17A producing CD4 + T cells in a dose-dependent manner (Fig. 4A, B). Furthermore, supernatants of the culture systems were collected and assayed for IL-17A and IFN-γ using ELISA. When compared with the blank control, cells stimulated by IRBP_1-20_ produced markedly higher levels of IL-17A and IFN-γ. However, the addition of melatonin mitigated IFN-γ and IL-17A production significantly (Fig. 4C, D). Since T-bet and RORγt are transcription factors for Th1 and Th17, we also examined the effect of melatonin on T-bet and RORγt. Our results demonstrated that melatonin suppressed T-bet and RORγt in vitro*.* (Fig. 4E, F). In addition, naïve CD4 + T cells were sorted and polarized under Treg conditions in the presence of melatonin or not. We found melatonin facilitated Treg cell differentiation dramatically (Fig. 4G). Together, these results supported the notion that melatonin can restrict IRBP_1-20_-specific T cell responses and promote Treg cell differentiation.

To further demonstrate the inhibitory ability of melatonin on the pathogenicity of IRBP_l-20_-specific T cells, we carried out an adoptive transfer experiment. The DLNs of EAU mice were isolated to make single cells suspension, which was stimulated by IRBP_1-20_ (10 μg/mL), separately, with a vehicle, or melatonin (200 ng/mL) under Th17-polarizing conditions for three days. Equal numbers of IRBP_1-20_-specific T cells pretreated with different treatment were adoptively transferred to the C57BL/6J mice via the tail vein. Two weeks later, we found melatonin treatment attenuated the disease severity (Fig. 4H). At the end of the experiment, the retinas histological analysis verified the clinical manifestation (Fig. 4I). These outcomes indicated that melatonin was able to regulate the effector functions of Th17 cells to alleviate EAU.

### Melatonin attenuated uveitis mainly by regulating the activation and functional status of CD4 + T cells

**Fig. 5:**
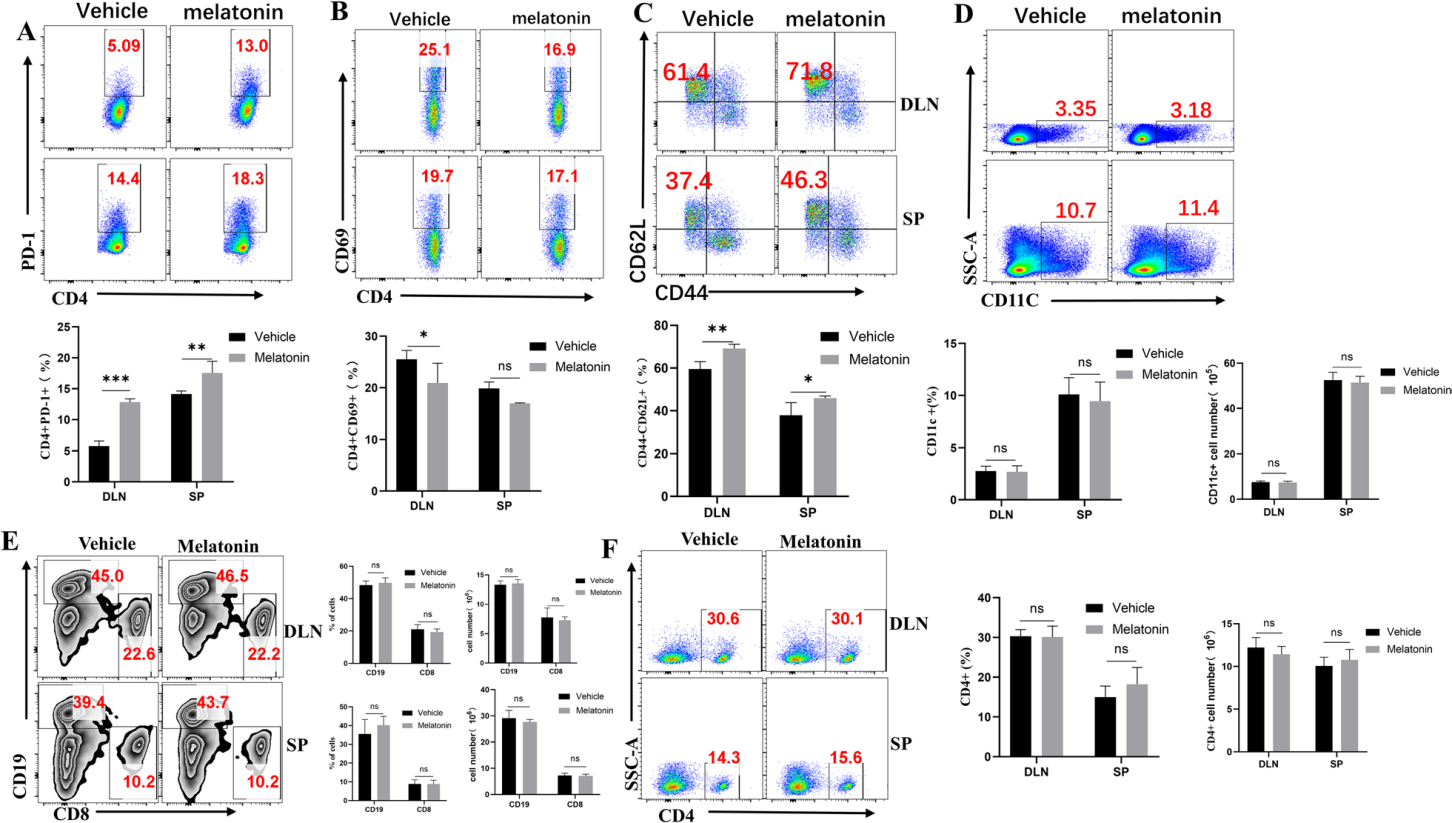
Melatonin attenuated uveitis mainly by regulating the activation and functional status of CD4 + T cells. **A**–**C** Frequencies of CD4 + PD-1 +, CD4 + CD69 + T cells, and CD44-CD62 + naïve CD4 + T cells from the spleen and DLNs of immunized mice in two groups. **D** Frequency and number of CD11C + cells from immunized mice in the vehicle-treated and melatonin-treated groups were measured by flow cytometry. **E**, **F** Frequency and number of CD8 + T cells, CD19 (B cells), and CD4 + T cells in immunized mice were measured by flow cytometry (four animals per group in three independently performed experiments). Significance was determined by two-way ANOVA (**A**–**F**). ^ns^*P* > 0.05, *P < 0.05, **P < 0.01, ***P < 0.001

Since EAU is a T cell-mediated autoimmune disease, we investigated whether melatonin could influence CD4 + T cells activation and function, and found that the expression of the T cell inhibitory receptor, programmed cell death protein 1 (PD-1), was significantly increased after melatonin treatment (Fig. 5A). To further investigate whether melatonin treatment inhibited lymphocyte activation, we analyzed the expression of the early activation marker CD69 on cells from spleen and DLN. Melatonin treatment significantly inhibited CD69 + cells in DLN, but not in the spleen (Fig. 5B). Subsequently, we analyzed naïve CD4 + T cells that were not activated by antigen during EAU. Interestingly, the percentages of  naïve T cells in the spleen and DLNs were much higher in the melatonin-treated group than in the vehicle-treated group (Fig. 5C).

As part of the innate immune system, dendritic cells (DCs) are the first to recognize and process antigens and present them to T cells to initiate autoimmune diseases. To ascertain whether melatonin treatment modulated DCs, we analyzed cells from DLNs and spleen of melatonin-treated EAU mice by flow cytometry. The frequency and number of DCs showed little difference between the two groups (Fig. 5D). Furthermore, flow cytometry results revealed negligible effects of melatonin on the frequency and number of CD8 + T cells, CD19 + B cells and CD4 + T cells (Fig. 5E, F). These data suggested that the therapeutic effect of melatonin in EAU was mediated mainly by the activation of CD4 + T cells.

### Melatonin suppressed Th17 cells from EAU mice via the oxidative stress/TXNIP/HIF-1α axis

**Fig. 6:**
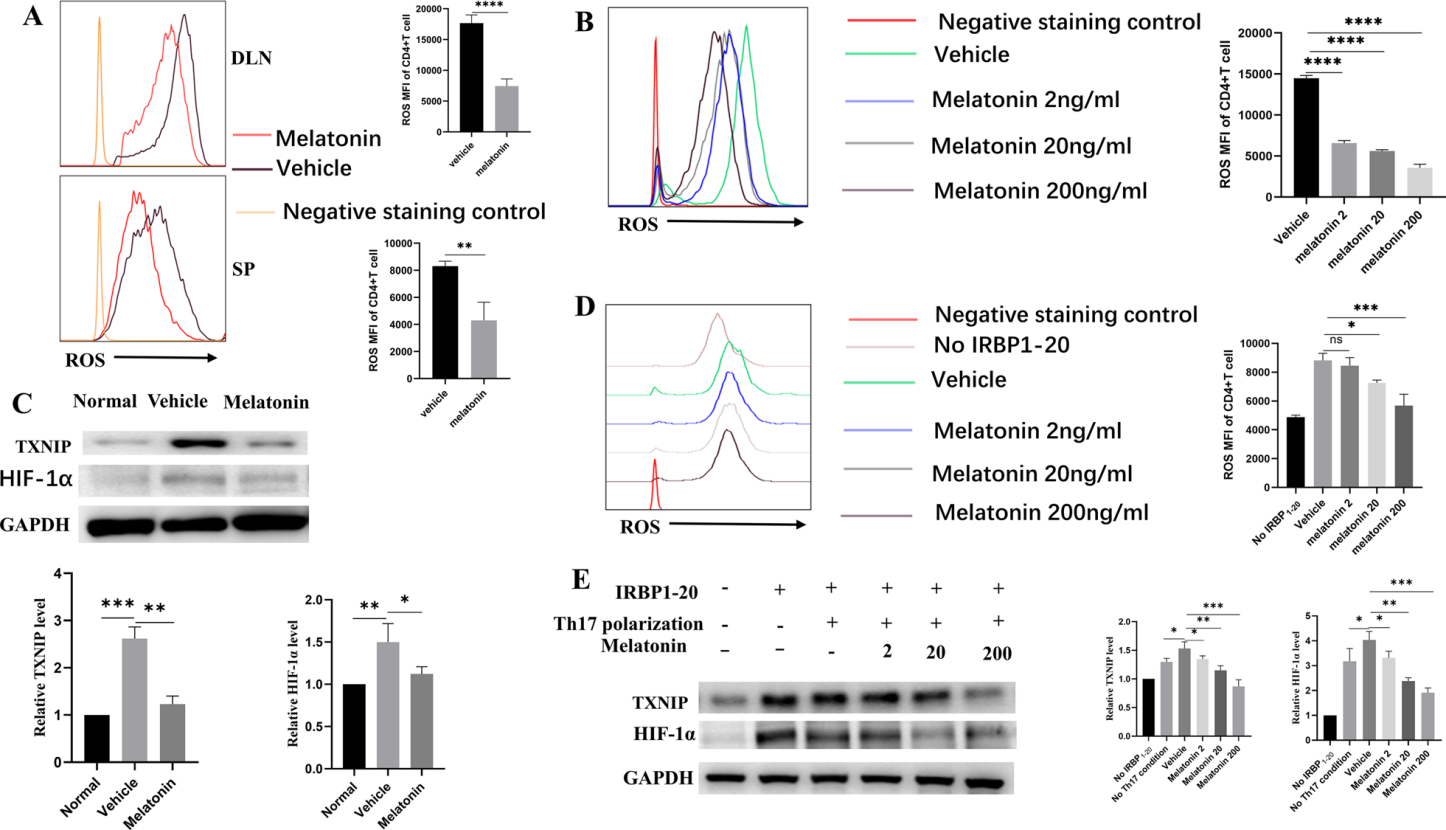
Melatonin suppressed Th17 cells from EAU mice via the oxidative stress/TXNIP/HIF-1α axis. **A** Reactive-oxygen species (ROS) MFI were analyzed by flow cytometry. **B** Effects of different concentrations of melatonin on ROS MFI during early activation of naive CD4 + T cells. **C** Western blot analyses TXNIP and HIF-1α in the CD4 + T cells from DLNs of EAU mice. **D** DLN cells from EAU mice were stimulated for 72 h with 10 μg/mL IRBP_1-20_ under Th17-polarizing conditions in the absence (vehicle) or presence of melatonin (2, 20, or 200 ng/mL). CD4 + T cells were assessed for ROS MFI by flow cytometry. **E** DLN cells from EAU mice were stimulated for 72 h with 10 μg/mL IRBP_1-20_ under Th17-polarizing conditions in the absence (vehicle) or presence of melatonin (2, 20, 200 ng/mL). Th17-polarized DLN cells was assessed for TXNIP and HIF-1α expression via western blot, respectively. The representative data from three independent experiments. Significance was determined by unpaired *t* test (**A**), or one-way ANOVA (**B**–**E**).*P < 0.05, **P < 0.01, ***P < 0.001

Reactive-oxygen species (ROS) are produced by enzymatic/nonenzymatic metabolic redox reactions, which can lead to various diseases and disorders, such as uveitis [21]. In particular, the redox protein thioredoxin (TRX) can produce free radicals, while TXNIP is an endogenous inhibitor of TRX. The TRX–TXNIP interaction plays a critical role in redox regulation and participates in cell proliferation and growth. Increased levels of ROS can disrupt the TRX–TXNIP homeostasis [5, 22, 23]. Previously research reported that downstream HIF-1α signaling could facilitate Th17 differentiation [24, 25]; so we investigated whether melatonin alleviated uveitis via the oxidative stress/TXNIP/HIF-1α signaling axis. We found that the levels of ROS MFI (Mean Fluorescence Intensity) in CD4 + T cells in the lymph nodes and spleen were significantly reduced in the melatonin treatment group (Fig. 6A). Further in vitro experiments also showed melatonin inhibited ROS as well as CD4 + T activation (Fig. 6B). EAU mice exhibited increased levels of TXNIP and HIF-1α. Meanwhile, the melatonin treatment greatly reduced TXNIP and HIF-1α levels (Fig. 6C). To verify the in vivo findings, we carried out in vitro experiments to determine whether melatonin could suppress ROS/TXNIP/HIF-1α and Th17 differentiation. DLN cells isolated from EAU mice were cultured with IRBP_1-20_ in the presence of melatonin or not under Th17-polarizing conditions for 72 h. ROS levels in CD4 + T cells were significantly reduced by melatonin in a dose-dependent manner (Fig. 6D). Furthermore, the expression of TXNIP and HIF-1α proteins were induced by IRBP_1-20_ stimulation, and attenuated by melatonin in a dose-dependent manner (Fig. 6E). Together, the above data supported the notion that melatonin suppressed Th17 cells from EAU mice via the ROS/TXNIP/ HIF-1α signaling axis.

## Discussion

In the present study, we probed into the beneficial effects of melatonin in EAU, a characteristic model of human non-infectious uveitis. Systemic administration of melatonin to mice inhibited the infiltration of inflammatory cells and facilitated Treg cells into the eyes. Melatonin could suppress the differentiation and function of uveitogenic effector T cells and potentiate regulatory T cells, which are crucial participants in the initiation and progression of uveitis. Additional mechanistic studies revealed that melatonin could effectively control disease progression by inhibiting the ROS/TXNIP/HIF-1α signaling axis.

Melatonin is produced mainly by the pineal gland and exerts multiple biological activities, including antioxidation, antiaging, and immunomodulatory properties, besides its circadian effect [26–28]. Melatonin has been proven to exert beneficial effects as an immune modulator in multiple autoimmune diseases, such as MS [8, 9] and inflammatory bowel disease (IBD) [29, 30]. However, the role of melatonin in patients with RA and in animal models of collagen-induced arthritis (CIA) remains controversial. Some studies suggested that melatonin has adverse effects in patients with RA and may increase the disease severity in the CIA model [31–33]. While other studies have documented the anti-inflammatory and immunoregulatory properties of melatonin. Tang et al. reported that melatonin mitigated the disease severity of rheumatoid arthritis by attenuating TNF-α and IL-1β expression in synovial fibroblasts and reducing cartilage degradation [34]. Korkmaz also advocated that melatonin was an appropriate adjunctive therapy for RA [35]. However, whether or not melatonin could effectively attenuate disease severity in patients with AU remains undetermined. Herein, we conducted experiments on mice and found that melatonin alleviated the severity of EAU.

It is worth noting that melatonin, as an endogenous hormone, is safe and nontoxic when secreted regularly in the body. Whether additional large doses and prolonged melatonin supplementation are harmful is debatable. In human studies, melatonin has been used to treat or prevent jet lag and tiredness at doses of 3–6 mg/day [36, 37]. López-González et al. reported that patients with primary progressive multiple sclerosis (PPMS) began taking only melatonin at doses ranging from 50 to 300 mg per day for 4 years, a higher dose melatonin could relieve symptoms more effectively without relevant adverse events[38]. In another melatonin clinical safety study, long-term high-dose melatonin (300 mg/ day) was administered through rectum for amyotrophic lateral sclerosis (ALS), and the clinical effects were satisfactory and well tolerated for 2 years. Therefore, it is suggested that high dose melatonin was suitable for the clinical trial of protecting the nervous system of ALS [39]. And in SOD1^G93A^ transgenic mice, high-dose oral melatonin delayed disease progression and extended survival. In many cancer animal models, high-dose melatonin significantly delayed the appearance and growth or metastasis of tumors [40, 41]. In EAE studies, melatonin dose was mostly used 5 mg to 200 mg/kg body weight [7, 8, 42, 43]. In our study, the actual cumulative dose of melatonin was 1.6 mg/ mouse (20 g)/day for 12 days, accumulating to 19.2 mg/ mouse. Indeed, many researchers worried about the adverse effects of high doses of melatonin, animal experimental studies have shown that melatonin could protect the liver [44], kidney, heart [45], and blood pressure [46] without obvious toxicity. Our study did not observe overt systemic toxicity (mainly evaluated by mortality rate, behavior, alterations in weight, liver, kidney, intestinal), consistent with the above researches (Additional file 1: Fig. S1).

Th17 cells have been reported to conduce to the pathogenesis of autoimmune diseases, such as MS, rheumatoid arthritis, IBD, and psoriasis, and were potential targets for immunotherapy [47]. Th17 also played a major pathogenic role in uveitis [48–50]. Researchers have shown that melatonin could influence the differentiation of Th17 and decrease Th17 infiltration in EAE models [6, 51]. Furthermore, melatonin treatment increased the percentage of Treg cells in the blood of patients with systemic lupus erythematosus and in EAE model [6, 8, 9, 52]. In addition, our results also found that melatonin could suppress auto-reactive Th17 cells and Th1 cells, but facilitate Treg cells both in vitro and in vivo*.*

As a potential candidate for the therapy of inflammatory diseases, whether melatonin had an effect on immune cells was a major concern. We evaluated the peripheral DC, T cell, and B cell populations of EAU mice treated with melatonin. Melatonin had no effects on the frequency of DCs in the spleen and DLNs. Furthermore, no obvious decrease was observed in the frequency of CD4 + T cells, CD8 + T cells, or CD19 + B cells after in melatonin-treated mice. Mauricio et al. confirmed that melatonin acted directly on CD4 + T cells rather than controlling them indirectly via DCs [8]. Considering that EAU was a CD4 + T cell-mediated disorder, and CD4 + T cell activation was the first step in initiating downstream events, we analyzed the effect of melatonin on CD4 + T activation, and found that the expression of both CD69 and CD44 were inhibited by melatonin. Alvarez-Sanchez N et al. reported that melatonin-treated mice significantly reduced the expression of CD44 [9]. Previous studies also confirmed melatonin influenced T cell-mediated immune responses, which were associated with membrane (MT1/MT2) and nuclear receptors (ROR, RZR), as well as receptor-independent pathways [51, 53–56]. Farez et al. have reported that melatonin could act on ERK1/2-C/EBPα/REV-ERBα via MTNR1 to suppress Th17 cell differentiation [8]. In addition, others have reported the melatonin-binding sites in the cytoplasm and mitochondria, including calmodulin, calreticulin, and quinone reductase-2 [57–59]. However, whether melatonin related receptors or receptor-independent pathways played a role in EAU remains to be elucidated.

ROS acted as a second messenger in T cell receptor signal transduction, which was essential for cell activation and effector functions [60, 61]. Previous studies have shown that biological stimuli induced cells to produce a large amount of ROS, and the increased levels of ROS disrupted the intracellular TRX–TXNIP balance [62]. TXNIP was a pivotal endogenous negative regulator of cellular redox balance [22, 63, 64] that was highly expressed in immune cells and played a role in regulating lymphocyte cycle progression and proliferation in the immune system [62, 65]. Sheng-Min Hsu, et al. have confirmed suppression of the ROS could alleviate EAU [21]. In addition, melatonin significantly attenuated ROS production in microglia to alleviate EAE [66]. Of note, Previte DM et al. reported that increased ROS in CD4 + T cells was associated with CD4 + T cell activation [67].We also verified that the melatonin reduced the levels of ROS in CD4 + T cells, as well as TXNIP, accompanied with the activation of CD4 + T cells. Melatonin could also control diabetic retinopathy via inhibiting expressions of TXNIP [5]. Inhibiting TXNIP in macrophages via reducing ROS could restrain DSS-induced colitis [68]. However, the effects of ROS/TXNIP redox metabolism on CD4 + T cell function and the mechanism of Teff/Treg immune homeostasis are not clearly revealed. Considering above, we further explored the HIF-1a expression during the process, which was reported to be greatly affected by ROS [69] and promoted Th17 differentiation through direct transcriptional activation of RORγt [24, 25, 70–72]. As expected, our results shown that melatonin significantly inhibited the increase of ROS and TXNIP expression in EAU mice, in addition to inhibiting the expression of HIF-1α. HIF-1α, a crucial metabolic sensor, regulated the balance between Treg and Th17 cell differentiation [25] and had a strong interaction with ROS/TXNIP, the down-regulation of which resulted in alleviation on EAU. Moreover, a study reported that melatonin could neutralize HIF-1α-controlled aerobic glycolysis through ROS cleaning up [73]. Therefore, our results contributed to the available literature by demonstrating the effect of melatonin on cellular immune balance from the perspective of the redox balance of CD4 + T cells.

## Conclusion

Taken together, our study demonstrated the therapeutic effect of melatonin on EAU for the first time. Melatonin significantly reduced local and systemic inflammatory responses and modulated T cell populations through the ROS/TXNIP/HIF-1α signal axis. These results not only enriched our understanding of the mechanism of EAU, but also expanded the clinical application potential of melatonin and offered a new and compelling option for the treatment of AU.

## Supplementary Information

**Additional file 1: Figure S1.** No evident signs of toxicity were observed in Melatonin-treated group. Melatonin(80 mg/kg/day) or (160 mg/kg/day) for 14 days. (A). Weight change was no significant difference between normal group, Melatonin 80mg/kg and 160mg/kg groups. (B). Representative H&E staining (Scale bar = 100 μm) sections of the liver and kidney after consecutive intraperitoneal injections of melatonin (80mg/kg, 160mg/kg) for 14 days. (C). Representative images about the changes of intestinal length in mice. n = 5 mice/group.

**Additional file 2: Figure S2.** Gating strategy for Fig2, and total numbers of infiltrating CD45+ cells in the eye. (A). Gating strategy for Fig 2(A-D), forward and side scatter gating on area gated lymphocyte, and excluded debris, then Fixable Viability Dye negative as live cells, subsequently CD45+, CD4+, CD4+IFN-γ+, CD4+IL17A+, CD4+Foxp3+ T cells were gated. (B). The number of eye-infiltrating CD45 + T cells were revealed by Flow cytometric analysis after melatonin treatment. The representative values from three independent experiments. Significance was determined by unpaired t test (n = 3), **P <0.01.

**Additional file 3: Figure S3.** Melatonin performed no significant effect on the proportions of Treg proliferation. Foxp3 + KI-67+T cells in the DLNs of EAU mice were evaluated by flow cytometry fourteen days after immunization (n = 4). The representative data from three independent experiments. Significance was determined by unpaired t test. ns P > 0.05.

## Data Availability

This article included all relevant data about this study.

## Abbreviations

AU: Autoimmune uveitis
EAU: Experimental autoimmune uveitis
ELISA: Enzyme-linked immunosorbent assay
HIF-1α: Hypoxia-inducible factor 1 alpha
IL-1β: Interleukin (IL)-1β
IFN-γ: Interferon gamma
IRBP_1–20_: Interphotoreceptor retinoid binding protein 1–20
PD-1: Programmed cell death protein 1
ROS: Reactive-oxygen species
RT-PCR: Real-time quantitative polymerase chain reaction
Th1: T helper 1
Th17: T helper 17
Treg: Regulatory T cells
Teff: Effector T cells
TXNIP: Thioredoxin-interacting protein
MFI: Mean fluorescence intensity
