# Supplementary figures and images for: Melatonin, an endogenous hormone, modulates Th17 cells via the reactive-oxygen species/TXNIP/HIF-1α axis to alleviate autoimmune uveitis
Source: J Neuroinflammation. 2022 May 27;19:124. doi: 10.1186/s12974-022-02477-z (PMC9145533; doi:10.1186/s12974-022-02477-z)

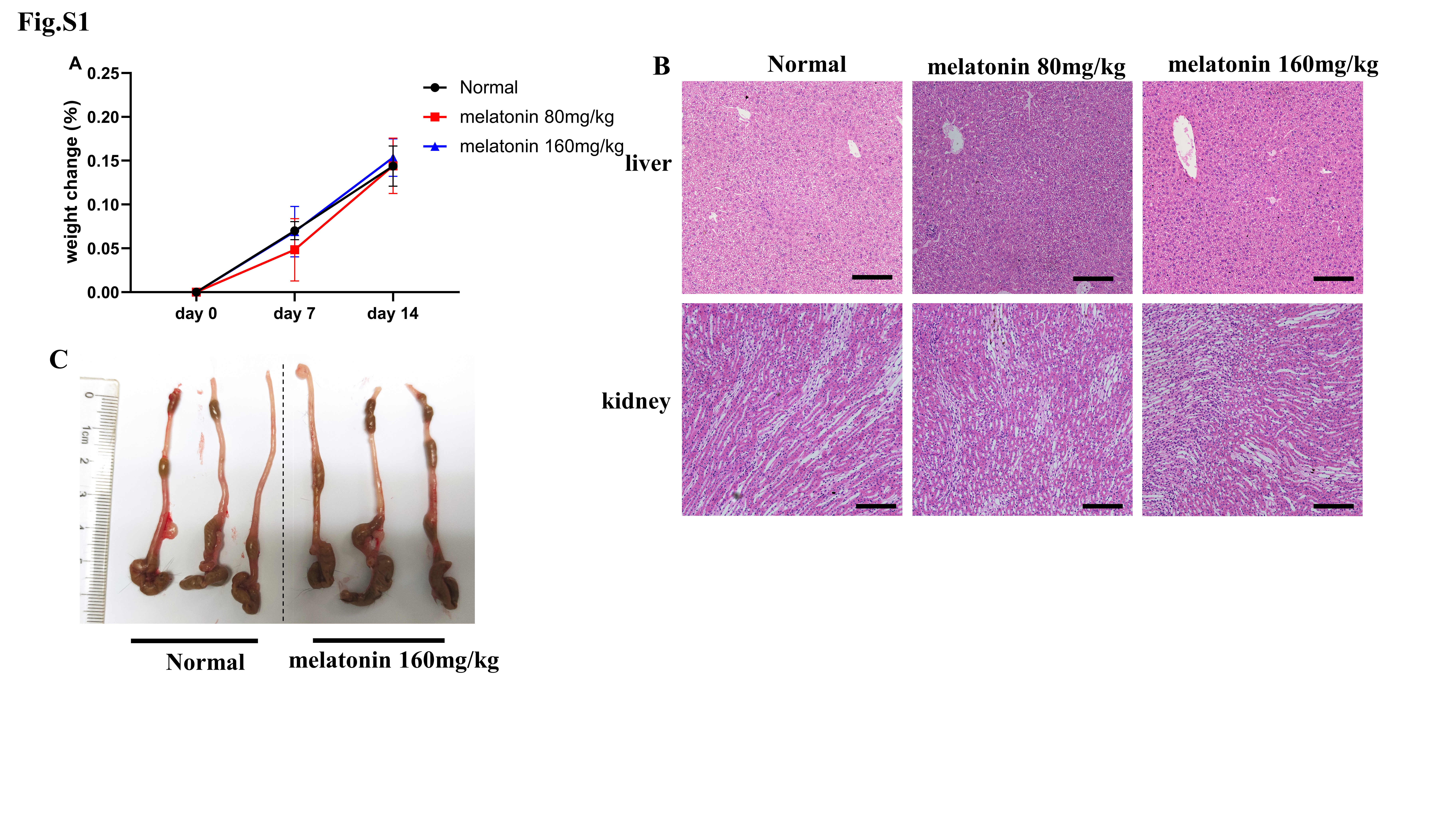

Supplement: Supplementary file 1 — Additional file 1: Figure S1. No evident signs of toxicity were observed in Melatonin-treated group. Melatonin(80 mg/kg/day) or (160 mg/kg/day) for 14 days. (A). Weight change was no significant difference between normal group, Melatonin 80mg/kg and 160mg/kg groups. (B). Representative H&E staining (Scale bar = 100 μm) sections of the liver and kidney after consecutive intraperitoneal injections of melatonin (80mg/kg, 160mg/kg) for 14 days. (C). Representative images about the changes of intestinal length in mice. n = 5 mice/group. [file 12974_2022_2477_MOESM1_ESM.tif]

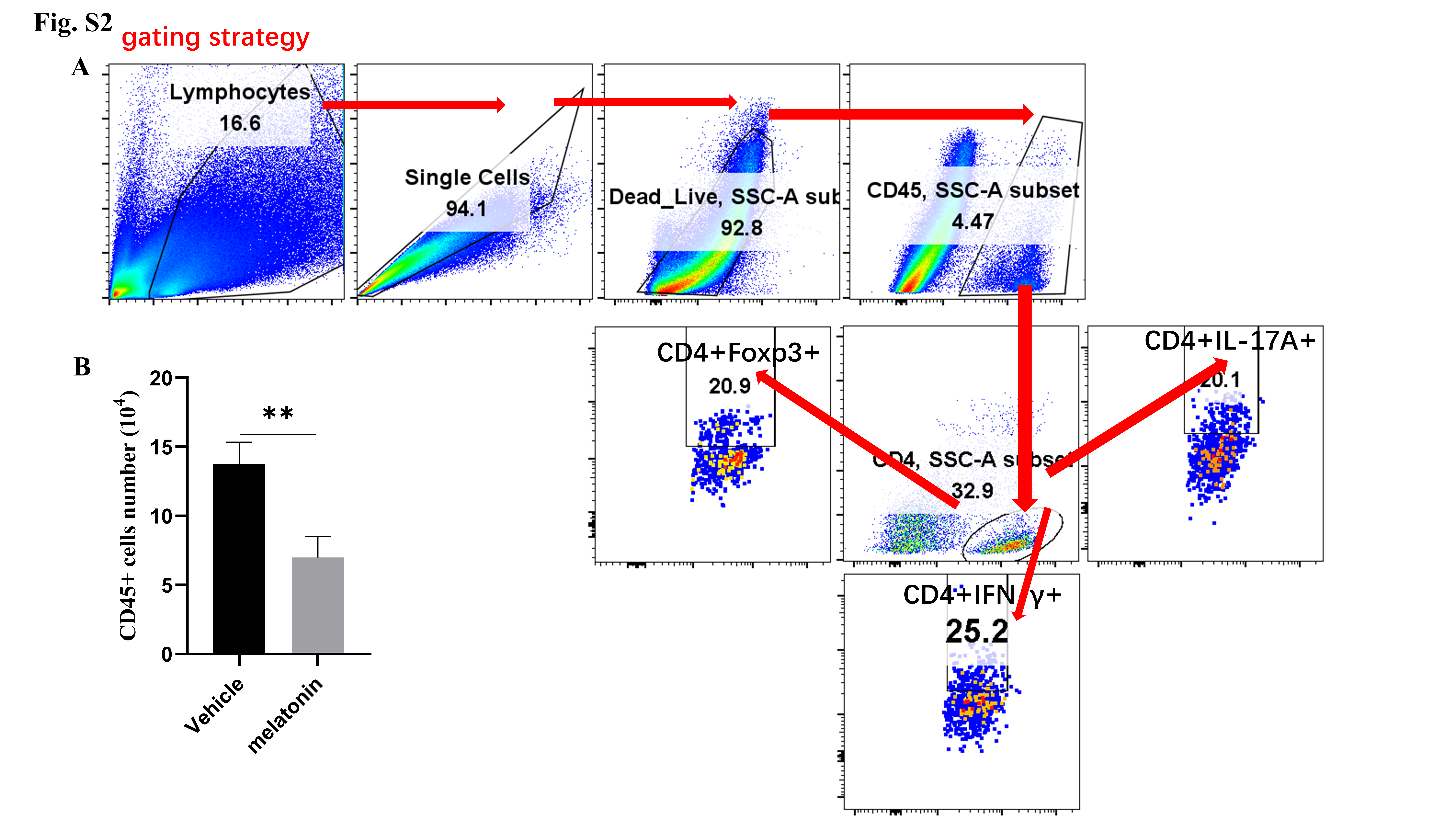

Supplement: Supplementary file 2 — Additional file 2: Figure S2. Gating strategy for Fig2, and total numbers of infiltrating CD45+ cells in the eye. (A). Gating strategy for Fig 2(A-D), forward and side scatter gating on area gated lymphocyte, and excluded debris, then Fixable Viability Dye negative as live cells, subsequently CD45+, CD4+, CD4+IFN-γ+, CD4+IL17A+, CD4+Foxp3+ T cells were gated. (B). The number of eye-infiltrating CD45 + T cells were revealed by Flow cytometric analysis after melatonin treatment. The representative values from three independent experiments. Significance was determined by unpaired t test (n = 3), **P <0.01. [file 12974_2022_2477_MOESM2_ESM.tif]

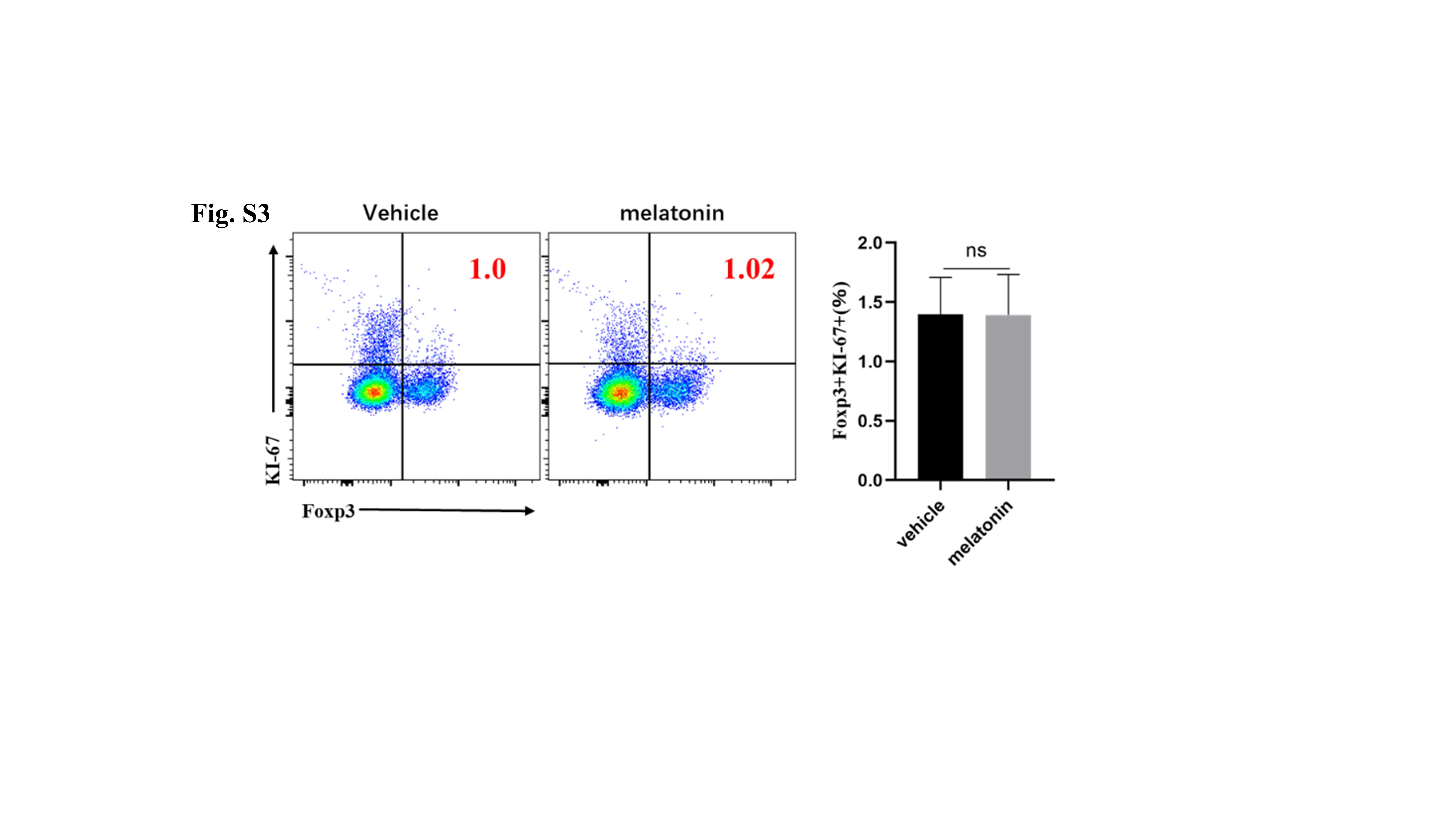

Supplement: Supplementary file 3 — Additional file 3: Figure S3. Melatonin performed no significant effect on the proportions of Treg proliferation. Foxp3 + KI-67+T cells in the DLNs of EAU mice were evaluated by flow cytometry fourteen days after immunization (n = 4). The representative data from three independent experiments. Significance was determined by unpaired t test. ns P > 0.05. [file 12974_2022_2477_MOESM3_ESM.tif]
